# Interactions between Schwann cell and extracellular matrix in peripheral nerve regeneration

**DOI:** 10.3389/fneur.2024.1372168

**Published:** 2024-03-26

**Authors:** Maorong Jiang, Muyang Chen, Nana Liu

**Affiliations:** ^1^School of Life Sciences, Key Laboratory of Neuroregeneration of Jiangsu and Ministry of Education, Co-innovation Center of Neuroregeneration, Nantong University, Nantong, Jiangsu Province, China; ^2^School of Pediatrics, Nanjing Medical University, Nanjing, China; ^3^The First School of Clinical Medicine, Lanzhou University, Lanzhou, Gansu, China

**Keywords:** axon biomaterials, extracellular matrix, myelin sheath, peripheral nerve regeneration, Schwann cells

## Abstract

Peripheral nerve injuries, caused by various reasons, often lead to severe sensory, motor, and autonomic dysfunction or permanent disability, posing a challenging problem in regenerative medicine. Autologous nerve transplantation has been the gold standard in traditional treatments but faces numerous limitations and risk factors, such as donor area denervation, increased surgical complications, and diameter or nerve bundle mismatches. The extracellular matrix (ECM) is a complex molecular network synthesized and released into the extracellular space by cells residing in tissues or organs. Its main components include collagen, proteoglycans/glycosaminoglycans, elastin, laminin, fibronectin, etc., providing structural and biochemical support to surrounding cells, crucial for cell survival and growth. Schwann cells, as the primary glial cells in the peripheral nervous system, play various important roles. Schwann cell transplantation is considered the gold standard in cell therapy for peripheral nerve injuries, making ECM derived from Schwann cells one of the most suitable biomaterials for peripheral nerve repair. To better understand the mechanisms of Schwann cells and the ECM in peripheral nerve regeneration and their optimal application, this review provides an overview of their roles in peripheral nerve regeneration.

## 1 Introduction

It is well-known that the nervous system possesses strong stability, and once damaged, spontaneous repair becomes extremely challenging (1). Peripheral nerve injuries are a common type of neurological damage in clinical practice, leading to varying degrees of autonomic dysfunction, sensory or motor impairments, localized paralysis, and chronic neuropathic pain, thereby diminishing patients’ quality of life (2). In the United States, the annual incidence rate of upper limb peripheral nerve injuries is 16.9 per 100,000 people, with an average emergency care cost of $5,779 per person. Calculated accordingly, the total annual medical expenses due to upper limb peripheral nerve injuries in the U.S. reach nearly $300 million (3). Therefore, peripheral nerve injuries represent a significant clinical and public health concern.

The causes of peripheral nerve injuries are complex, including cutting injuries, tearing injuries, traction injuries, compressive injuries, and iatrogenic injuries. Among these, traction-related peripheral nerve injuries are the most common, followed by tearing injuries and compressive injuries that can cause complete transverse damage to the affected nerves (4). Clinically, the Seddon or Sunderland classification methods are often employed. Seddon classifies the severity of nerve injuries into neurapraxia (temporary functional changes with preserved axonal continuity), axonotmesis (loss of axonal continuity with varying degrees of endoneurial disruption, temporary loss of function), and neurotmesis (complete loss of axonal and endoneurial continuity, incomplete nerve regeneration, and incomplete functional recovery) (5). Sunderland refines the Seddon classification into five degrees, corresponding to varying degrees of severity from concussion (first degree) to rupture (fifth degree), providing a more detailed assessment of overall injury severity and recovery difficulty (4).

Different nerve repair methods are adopted based on the varying degrees of nerve injury. Autologous nerve transplantation is the most widely used bridging technique for repairing peripheral nerve defects, currently considered the “gold standard” for treating long-distance peripheral nerve defects (6, 7). However, autologous nerve transplantation comes with numerous limitations and risk factors, such as denervation of the donor area, increased surgical complications, and diameter or nerve bundle mismatches. With the development of tissue engineering, tissue-engineered nerve grafts (TENG) have become an important potential alternative to autologous nerve grafts (8).

The ECM is a complex molecular network synthesized and released into the extracellular space by cells residing in tissues or organs (9). Its main components include collagen, proteoglycans/glycosaminoglycans, elastin, laminin, fibronectin, etc., providing structural support and attachment sites for cells (10). Schwann cells, as the primary glial cells in the peripheral nervous system, play various crucial roles and are considered the gold standard in cell therapy for peripheral nerve injuries (7, 11). Therefore, ECM derived from Schwann cells has become one of the most suitable biomaterials for peripheral nerve repair (7). To explore new approaches for peripheral nerve injury repair, this article provides a review of the key histological foundations of peripheral nerve regeneration - Schwann cells and ECM, as well as their interactions.

## 2 Neural repair after injury

Although the peripheral nervous system possesses strong regenerative potential, spontaneous regeneration rarely occurs in cases of nerve transection, and the outcomes of treatment interventions are often unsatisfactory (12). Conservative treatment and surgical intervention are the two main approaches for peripheral nerve injury (2). Conservative treatment involves non-surgical methods to protect damaged neurons, promote nerve regeneration and maturation, thereby facilitating the recovery of nerve function (13). Surgical treatment, depending on the type and severity of peripheral nerve injury, may employ direct nerve anastomosis or bridging repair as two repair strategies (14). For injuries with a small nerve defect distance (5 mm), surgical suturing is generally considered the best repair method, while nerve transplantation is required for larger defects (≥5 mm) (15). Autologous nerve transplantation is the most widely used bridging technique in clinical practice for repairing long-distance peripheral nerve defects and is currently considered the “gold standard.” Autologous nerve transplantation has advantages such as good regeneration effects (higher vascularization, an elevated degree of anastomosis), minimal immune rejection impact, etc. However, it comes with inevitable drawbacks, including limited availability of autologous nerve grafts, denervation of the donor area, nerve tumor formation, increased infection risk, diameter mismatch, etc. Therefore, optimization and exploration of alternative solutions are crucial (16–21).

In recent years, with the advancement of tissue engineering research, tissue-engineered nerve grafts are considered promising alternatives to autologous nerve grafts (22). Tissue-engineered nerve grafts encompass tissue-engineered neural biomaterial scaffolds, mainly including nerve conduits and their fillers, with diverse structural forms. They can be tailored to repair peripheral nerves of different diameters and defect lengths, making them a current hotspot in peripheral nerve repair and regeneration (23). Tissue-engineered nerves consist of three elements: scaffold, seed cells, and soluble regulatory factors. The core idea is to use living cells to combine with ECM or scaffold material in a certain way, and add factors that induce and promote growth to form neural tissue *in vitro*, replacing damaged tissue (24).The neural scaffold or conduit provides a foundational space for nerve regeneration, guiding axonal regeneration and preventing scar formation. However, seed cells and soluble regulatory factors face various limitations. Therefore, there is an urgent need to find an alternative biomaterial that can support cells and growth factors, leading to the construction of a novel tissue-engineered nerve graft (25). ECM possesses various characteristics, promoting axonal regeneration while avoiding the limitations of supporting cells and growth factors. Hence, it holds the potential to replace seed cells and growth factors, becoming a crucial biomaterial in the construction of innovative tissue-engineered nerves (26, 27).

## 3 Significance of Schwann cells

Undoubtedly, Schwann cells play a dominant role in the regeneration and development following peripheral nerve injuries (16, 28–32). Derived from the neural crest, Schwann cell precursors (SCPs) can generate various polarized cells, including Schwann cell precursors (33, 34). These precursors proliferate rapidly, developing into immature SCs, which subsequently differentiate into two mature types of SCs: myelinating SCs and non-myelinating SCs (35). In recent years, scholars have discovered another subset of mature Schwann cells (SCs), termed repair Schwann cells, which are formed through adaptive cell reprogramming following nerve injury (36). Although non-myelinating SCs do not produce myelin, they still play important roles in the peripheral nervous system, such as maintaining axonal metabolism and preventing neuropathic pain. Non-myelinating SCs still possess the potential for myelination. After axonal or nerve injury, SCs trigger defense mechanisms involving inflammation and immune responses, activate Wallerian degeneration, and promote the survival of damaged neurons and axon regeneration (7). At this stage, both myelinating and non-myelinating SCs transition to repair Schwann cells to initiate the repair process (37). This phenotypic change requires adaptive cell reprogramming of SCs, including dedifferentiation of myelin and activation of axon repair and regeneration functions (36). These characteristics indicate that SCs are highly plastic and can undergo conversion between different subtypes in response to environmental signals.

Recent studies have found that Schwann cells have functions such as migration, adhesion, ECM production, secretion of various neurotrophic factors, and bioactive substances (38, 39). Neurotrophic factors include NGF, BDNF, CNTF, FGF, NT, GAP-43, etc. (40). Secreted ECM components include FN, LN, IV collagen, V collagen, heparin sulfate proteoglycan (HSPG), and entactin (38). These ECM components are deposited outside Schwann cells to form the basal lamina. Schwann cells also synthesize cell adhesion molecules (CAMs), which affect cell adhesion. CAMs include neural cell adhesion molecule (N-CAM), neural glial cell adhesion molecule (NG-CAM), MAG, peripheral myelin protein, etc. ECM and CAMs, in the early stages of nerve regeneration, regulate the initial extension of nerves, growth rate, and maturity, promote cell adhesion, maintain the stability of growth cone advancement, and accelerate axonal initiation and growth (41).

Myelinating Schwann cells, the focus of this discussion, exhibit radial and longitudinal polarization. As myelination progresses, Schwann cells organize into distinct domains, each with a unique protein array and a set of interconnected cytoplasmic compartments. Longitudinal polarity is prominently manifested in the overall organization of myelinating Schwann cells and axons, forming distinct nodal, paranodal, and internodal compartments. Radial polarity is evident on different inner and outer membrane surfaces, present at opposite ends of the cell; the compact myelin sheath inserts between these two domains (42). Myelinating Schwann cells express many typical genes in an immature state under normal physiological conditions and many newly expressed genes (such as EGFL8 gene, H3K27 gene, Oct6 gene, and Krox20 gene). These genes regulate and drive the process of neuron survival, damaged axon breakdown, and the regeneration process. The regeneration process involves myelin clearance, axon regeneration, guiding regenerated axons to their normal physiological locations, and ultimately remyelinating the regenerated axons (35, 43, 44). Therefore, elucidating the various biochemical mechanisms influencing Schwann cell growth, proliferation, function, and apoptosis is undeniably crucial for peripheral nerve regeneration research.

## 4 Schwann cell-related growth factors and pathways

Macrophages secrete interleukin (IL)-1 after injury, inducing Schwann cells to release neurotrophic factors and transforming them into a regenerative phenotype. The jun gene transcription product c-jun plays a crucial role in this process, regulating the expression of 172 genes in Schwann cells. Prolonged denervation after injury leads to a decrease in the expression of this key factor, affecting Schwann cell phenotypic differentiation. Therefore, one potential direction is to add chemical stimulants to scaffolds to upregulate c-jun expression, thereby activating more Schwann cells with the Remak Schwann cell (RSC) phenotype (30, 45). Research has shown that the guidance receptor Plexin-B2 also plays a crucial role in this process. The expression of this receptor is significantly upregulated in infiltrating macrophages of damaged nerves, and it can guide the alignment of macrophages and Schwann cells, thereby preventing collisions with axons. Conditional deletion of Plexin-B2 in the myeloid lineage not only leads to misplacement of macrophages but also results in matrix disarray and Schwann cell disintegration, consequently causing axonal misguidance and delayed functional recovery (46). Additionally, another critical cellular factor, Exendin-4, has been found to promote Schwann cell proliferation and migration by activating the Jak–STAT pathway, thereby effectively promoting the repair process after nerve injury (31).

Research has focused on improving the efficiency of basic fibroblast growth factor (bFGF) transmission in the process of nerve regeneration. bFGF is a crucial growth factor for nerve injury repair, promoting Schwann cell proliferation. In one study, a specific collagen-binding domain (CBD) was fused to the N-terminus of bFGF. This fusion complex was then combined with a linearly ordered collagen fiber scaffold. The modified scaffold was transplanted into the cut end of the rat sciatic nerve, effectively enhancing bFGF factor transmission efficiency and regeneration outcomes (30). Tomokazu Fukuda and colleagues combined a controlled release system of basic fibroblast growth factor (bFGF) with biodegradable nerve conduits. They used enzyme-linked immunosorbent assay (ELISA) to measure the release of bFGF and investigated its effects on neurovascularization and Schwann cell proliferation in a mouse sciatic nerve model. The results indicated that compared to the control group, the experimental group with the addition of the controlled release system exhibited slow release of bFGF, leading to improvements in neurovascularization and Schwann cell proliferation (47).

It has been found that beta-site amyloid precursor protein cleaving enzyme 1 (BACE1) weakens Schwann cell-mediated peripheral nerve regeneration. Inhibiting its activity can promote muscle nerve redenervation (48). MicroRNA (miRNA) is a short endogenous RNA with the potential to regulate and silence the expression of any RNA. Injecting miRNA into ECM during the regeneration process to silence the mRNA encoding BACE1 in Schwann cells might promote nerve regeneration and muscle nerve redenervation.

Osteopontin (OPN) is a glycoprotein containing the RGD (arginine-glycine-aspartic acid) sequence, exhibiting cytokine-like, chemotactic, and pro-adhesive properties. It is expressed in Schwann cells in the degenerated distal nerve stump and is regulated by axon-derived signals, significantly reducing severe axonal multifocal neuropathy (49). OPN is crucial for the secretion of type I collagen during dentin repair, improving the microenvironment for dental nerve regeneration (50). Application of OPN in the healing of replanted mouse teeth resulted in reduced inflammation and better reinnervation of blood vessels and nerves compared to the control group (51).

The main challenge in Schwann cell research is the inability to determine to what extent *in vitro* cultures of human Schwann cells reflect the changes and development of Schwann cell phenotypes in the actual human peripheral nerve environment after injury. This awaits the emergence of new research methods.

## 5 ECM and its connection with Schwann cells

### 5.1 Overview of ECM structure in the Schwann cell environment

The normal growth of Schwann cells relies on a well-established ECM environment. All tissues and organs contain a mixture of cells and non-cellular components, where the non-cellular component forms a well-organized network known as ECM. The ECM is composed of various matrix macromolecules, and its precise composition and specific structure vary between tissues. The main components include fibrous proteins such as collagen, elastin, fibronectin (FN), laminin, glycoproteins, proteoglycans (pg), and glycosaminoglycans (GAGs), which are highly acidic and hydrated molecules. This extensive meshwork system provides structural support to cells and regulates intercellular communication. The 3D arrangement of cells through the ECM creates physical paths for cell movement. Additionally, ECM can interact with various molecules, such as growth factors, signal receptors, and adhesion molecules, influencing a range of cellular behaviors and functions, including cell growth, migration, differentiation, survival, homeostasis, and morphogenesis (52–54). Undoubtfully, this interaction is particularly crucial for axon regeneration and the redirection of nerve fibers.

Next, we will provide an overview of the major components of ECM and their impact on Schwann cell growth.

#### 5.1.1 Collagen

Collagen is the most abundant protein in mammals. The collagen family includes 28 members, each containing at least one triple helical structure domain. Collagen is deposited in the ECM, where most form supramolecular assemblies, aiding in tissue organization and shaping. Collagens also interact with cell surface receptors, regulating cell proliferation, differentiation, and migration. In the differentiation process of peripheral nerves, Schwann cells predominantly express a member of the V-type collagen family, α4(V) collagen. This collagen protein has high affinity with heparan sulfate through a unique binding motif in the non-collagen N-terminal domain (NTD). The primary α4(V) collagen-binding protein on the Schwann cell surface is heparan sulfate proteoglycan-1. In co-culture, α4(V) collagen binds with fibronectin on the polarized Schwann cell surface, forming tubular ECM structures, crucial sites for myelin sheath formation. Inhibiting glypican-1 or alpha4(V) collagen expression significantly inhibits myelin sheath formation, highlighting their critical role in peripheral nervous system myelination. Furthermore, it has been found that type VI collagen is crucial for the migration and polarization of macrophages during peripheral nerve regeneration. Nerve injury induces a strong upregulation of type VI collagen. *In vitro* studies have demonstrated that type VI collagen promotes macrophage migration and polarization through the AKT and PKA pathways, regulating macrophage function and consequently modulating peripheral nerve regeneration (55–57).

#### 5.1.2 Elastin

Elastin is a crucial, long-lived ECM protein primarily found in major arteries, lungs, ligaments, tendons, skin, and elastic cartilage. Secreted by the Eln gene, elastin is organized into elastic fibers, providing elasticity and resilience to various vertebrate tissues. Elastin represents a class of heat-triggered phase separation thermosensitive peptide polymers with a low critical solution temperature, exhibiting good tissue compatibility (58, 59). Elastin can enhance the elasticity and flexibility of myelin sheaths after peripheral nerve regeneration.

#### 5.1.3 Fibronectin

Fibronectin (FN) is a 500 kDa dimer glycoprotein with variable molecular conformations and splice variants. Each FN molecule consists of three types of repeating subunits. FNI and FNII are stabilized by disulfide bonds in beta-sheets, while FNIII is a mechanically deformable seven-stranded beta-barrel structure. The FNIII9-10 domain contains the synergy-binding site (PHSRN) and the RGD-binding site, regulating integrin adhesion. FN can be classified into various types based on molecular conformation and splice variations, preferentially binding to cells through integrins, other FN subunits, collagen, heparin, fibronectin, matrix metalloproteinases (MMPs), and growth factors. FN is classically divided into two types: plasma-soluble and cellular (60). In the peripheral nervous system, fibronectin is primarily secreted and produced by Schwann cells, sparsely distributed along the cell surface, and mainly located within the perineurium (61).

Fibronectin binds to the cell surface through receptor-ligand interactions and transmits signals into the cell, thereby mediating the growth of neuronal axons (62). *In vitro* experiments have demonstrated that fibronectin can promote Schwann cell proliferation and stimulate their directed migration and chemotaxis (63). During the early stages of peripheral nerve regeneration, fibronectin has been observed to regulate axonal growth and positively influence crucial cells involved in nerve repair, including Schwann cells and macrophages (64).

In the peripheral nervous system, the interaction between neurons and fibronectin is primarily mediated by β1 class integrin heterodimers. During neural development, fibronectin and α5β1 integrin are expressed at relatively high levels in neurons (65). In mature nerve injuries, both fibronectin and α5β1 integrin are upregulated. Therefore, it can be observed that fibronectin and its receptor α5β1 may mediate functionally important interactions during the development and regeneration of the peripheral nervous system (66). α5 integrin is localized in adhesion complexes within the growth cones and protrusions of injured peripheral nerves, and injured sensory nerves exhibit a stronger response to fibronectin compared to normal nerves (67). Thus, in injured nerves, enhanced axonal growth mediated by fibronectin is largely facilitated by α5β1 integrin.

Studies by Mosahebi et al. suggest that adding fibronectin to a bioengineered nerve conduit scaffold based on alginate hydrogel promotes nerve regeneration, supports Schwann cell vitality, and enhances its influence on axon growth during nerve conduit transplantation, emphasizing its crucial role in axon regeneration (68).

#### 5.1.4 Laminin

Laminin is a high-molecular-weight glycoprotein composed of three disulfide-bonded peptides, α, β, and γ chains. The human genome encodes 11 genes for different laminin chains. Different laminin isoforms can bind to different molecules, and laminin is present on all basement membranes. Laminin can interact with type IV collagen, nidogen, perlecan, and heparan sulfate proteoglycans, assembling into the basement membrane (69, 70). A common and crucial function of laminin is its interaction with receptors anchored on the cell membrane near the basement membrane. In this process, laminin regulates various cellular activities and signaling pathways. Structurally, laminin consists of several independently folded, distinct domains, the number, location, and size of which, as well as their interaction with other molecular components of the basement membrane, vary among laminin members (71). It has been found that in models of nerve regeneration after peripheral nerve injury, the additional addition of cross-linked laminin complexes can lead to the observation of many new capillary-like structures around regenerating nerves, suggesting its potential to promote nerve regeneration by inducing angiogenesis (72).

#### 5.1.5 Glycoproteins and proteoglycans

These components have diverse compositions and are not detailed here.

#### 5.1.6 Glycosaminoglycans

Glycosaminoglycans (GAGs) are long linear polysaccharides composed of repeating disaccharide units, typically consisting of uronic acid and amino sugar. The repeating disaccharide units, except for keratan sulfate, where glucuronic acid is replaced by galactose, consist of a sugar aldehyde and an amino sugar (73). GAGs are present in vertebrates, invertebrates, and bacteria (15). Due to their high polarity, GAGs attract water, serving as lubricants or shock absorbers in the human body. In the ECM environment after peripheral nerve injury, GAGs may play a role in shaping and guiding Schwann cell growth. A study suggests that incorporating a synthetic collagen substrate matrix containing chondroitin sulfate 6 into the lumen significantly improves bridging and functional recovery in a rat model of nerve injury (74).

### 5.2 Scaffold material selection

The materials used to construct nerve scaffold conduits can be classified into two major categories: natural and synthetic. Compared to synthetic materials, natural materials exhibit higher compatibility, faster degradation, non-toxic degradation products, and the ability to provide cell adhesion factors and their binding sites, making them more promising for applications. To promote the structural and functional repair and regeneration processes after peripheral nerve injury, exploring and improving natural biomaterials that closely mimic the highly directional native ECM can be crucial. These materials can bridge the gap in injured nerves, provide guidance for regenerating axons, and support a protective microenvironment, thereby facilitating better cell growth and reducing the probability of nerve tumor formation. The closer the microstructure of a good nerve scaffold conduit material is to natural tissue, the greater the success rate of regeneration transplantation (10, 29, 75–77).

As mentioned earlier, collagen is the most abundant protein in humans and animals, accounting for 25–35% of total proteins. It is also a major inherent component of the ECM in peripheral nerves, playing a crucial role in nerve development and maintenance of nerve function. Collagen’s fibrous structure, close to natural nerve tissue, and its better revascularization effects make it an important alternative material. It can deposit in the ECM of Schwann cell basement membranes and peripheral nerves to provide structural support, and through surface receptors, aid in myelin regeneration and functional recovery. However, collagen has the disadvantage of poor mechanical and physical properties (75, 78, 79).

To improve its performance for better scaffold construction and transplantation, attempts have been made to modify collagen by adding SiO2. The results showed a significant reduction in the scaffold’s porosity, swelling rate, and degradation rate, and a notable improvement in mechanical and physical properties. Schwann cell viability and DNA content increased initially with SiO2 concentration and then declined. The optimum concentration for the lowest cytotoxicity was found to be 25 μg/mL [59]. Another approach involves combining collagen with chondroitin sulfate using electrospinning technology to build a scaffold. The fiber orientation of the scaffold was observed under scanning electron microscopy to assess its impact on cell tissue growth. The results showed that the fiber orientation and tensile strength of the scaffold were close to normal nerve tissue. Additionally, using a centrifuge produced more orderly oriented fibers (75).

Some researchers proposed using nanosilver particles combined with collagen to significantly increase the adsorption capacity of fibronectin (a crucial protein for stabilizing nerve morphology and adsorbing seed cells) and achieve better morphological and functional recovery. It was observed that in the animal model of 10 mm peripheral nerve injury, compared to the control group without nanosilver, the silver-containing group had thicker myelin, faster conduction speed, and higher maximum nerve potential. Moreover, it could avoid the negative effects of the traditional polyglycolic acid (PGA) scaffold’s degradation process, which leads to a decrease in pH and further damages the residual scaffold structure, triggering a positive feedback effect that accelerates degradation and harms the cell microenvironment. The team successfully used type I collagen combined with nanosilver to promote the regeneration of rabbit sciatic nerves (29). In addition to collagen, chitosan is another alternative material in the field of nerve regeneration. Chitosan is derived from chitin extracted from crustaceans, insects, and mushrooms. Similar to collagen, it exhibits good biocompatibility but has limitations in terms of plasticity and mechanical properties. The binding of chitosan with Schwann cells can increase the probability of damaged nerve connections, producing more ECM components similar to natural scaffolds (mainly collagen, proteoglycans/glycosaminoglycans (PG/GAGs), elastin, etc.). This stimulation further induces the secretion of fibronectin, a key protein in ECM that regulates tissue homeostasis and controls core cell processes and participates in the regulation of axon myelination. This secretion can lead to the formation of vascular basement membranes and peripheral myelinated axon regeneration. Chitosan’s unique physicochemical composition allows it to mimic the physiological multilayer structure of peripheral nerves, making it suitable for biomimetic structures (80–82).

Researchers led by Florian Neubrech attempted to use chitosan nerve conduits to repair acute human hand sensory nerve injuries. The results showed that the additional use of chitosan nerve conduits significantly reduced the occurrence of painful neuromas. Moreover, there was a correlation between clinical improvement in function (static two-point discrimination ability) and the use of chitosan conduits (83). The team led by Yuval Shapira conducted a comparative study between chitosan hollow tubes and autologous nerve grafts. After 90 days, there were no statistically significant differences in electrophysiological indices, muscle function, and morphology-related indicators between chitosan hollow tubes and autologous nerve grafts, suggesting its potential application prospects (84). Rachel Sarabia-Estrada and colleagues implanted chitosan nerve conduits loaded with progesterone to regenerate the sciatic nerves of rats. The results indicated that, after 90 days, rats with chitosan conduits containing progesterone showed better recovery in knee joint angle displacement, stride length, movement speed, and hindlimb lift-off the ground compared to the regular chitosan group (85). Chitosan biodegradation can form chito-oligosaccharides (COS), which possess neuroaffinity and neuroprotective effects. Yanpei Gong found that COSs can accelerate peripheral nerve regeneration after rabbit sciatic nerve crush injury (86).

Apart from collagen and chitosan, some niche materials are worth mentioning. A team led by A E Carolus used bovine pericardium as a wrapping material for nerve scaffolds transplanted to patients with peripheral nerve injuries. The results showed improvement in both function and pain for all patients. The material used showed no adverse reactions, and compatibility tests indicated seamless integration with the environment without causing nerve reformation scars, suggesting that bovine pericardium is a promising allogeneic material for nerve wrapping (87).

### 5.3 Improvement in the manufacturing process of nerve conduit scaffolds

In the context of nerve conduit scaffolds, material selection is not the only crucial factor; the manufacturing process also plays a key role in determining the final performance and effectiveness. Traditional processes, including electrospinning, freeze-drying, and centrifugal casting, have limitations in replicating the structure, fiber alignment, and physicochemical parameters of natural ECM. A newer production process for scaffolds, high-resolution 3D printing, has entered the animal experimentation stage. The scaffolds produced using this method demonstrate superior effects in promoting axonal directional growth and myelin sheath formation compared to simple cell transplantation. This is attributed to the ability of 3D printing to generate a complex and intricate internal structure that closely resembles natural ECM. The goal of 3D printing in peripheral nerve regeneration is to automate the manufacturing of structures inside nerve conduits, potentially rivaling autologous nerve grafts in cases of large gap injuries, and allowing for precise customization for patients (16, 88).

Another approach to improving the ECM condition after nerve conduit transplantation involves constructing acellular nerve grafts (ANG) with ECM derived from homologous dental pulp stem cells (DPSCs). In ANG, cells and myelin from DPSCs are removed, preserving the original ECM. This promotes Schwann cell attachment and proliferation without triggering a strong immune response. However, the effectiveness of ANG is still weaker than that of autologous nerve transplantation (89).

The products obtained after decellularization of neural tissue and processed through specific procedures to create tissue-derived ECM and applied in neural regeneration research are also of significant importance (9, 27). Chen et al. prepared decellularized ECM and polydopamine (PDA)-coated 3D-printed poly(ε-caprolactone) (PCL) conduits. The results showed that dECM/PDA-coated PCL conduits exhibited favorable mechanical properties compared to nerves from humans or animals. The dECM/PDA-coated PCL nerve conduits could serve as a practical and clinically feasible tool to promote the regeneration of longer peripheral nerve defects (90).

From the above studies, it is evident that nerve conduits are moving toward greater diversity in materials, combining organic and inorganic components. The manufacturing process is becoming more sophisticated, gradually approaching the goal of creating an environment similar to natural ECM. This direction aims to provide an environment more conducive to the growth and proliferation of Schwann cells, ultimately improving the success rate of nerve regeneration.

### 5.4 Complex interactions between ECM and Schwann cells

Further research is underway to explore the intricate interactions between ECM and Schwann cells (SCs). The team led by Peng Yu found that laminin (LN), fibronectin (FN), and type IV collagen (IV-Col) possess the ability to promote early adhesion of SCs in 2D culture. However, there are significant differences in the proportion of early cell adhesion, and the expression levels required for maintaining cell morphology vary markedly with different ECM proteins (10). Stephanie J Armstrong’s earlier study on the impact of ECM molecules on SC adhesion and proliferation on poly(3-hydroxybutyrate) (PHB) nerve conduit material yielded similar findings, highlighting the role of LN in enhancing synaptic growth by activating NF-kappaB in SCs (91, 92). Taogen Gong’s team, using single-cell RNA sequencing (scRNA-seq), revealed strong interactions between ECM and SC-related subgroups, possibly mediated by the SEMA3C signaling pathway and MK/PTN gene family, which are crucial for promoting SC proliferation and migration, thus facilitating earlobe scar nodule formation (93). Regulating and activating these pathways during peripheral nerve regeneration can accelerate SC proliferation and migration, promoting the regeneration process.

Xu and colleagues analyzed the impact of ECM hardness and cell morphology on SC plasticity, finding that increased ECM hardness and SC spreading downregulated regenerative proteins through the activation of Rho GTPase and YAP/TAZ. At the same time, cell elongation promoted unique SC regenerative abilities through the upregulation of Rac1/MKK7/JNK, essential for the ECM and morphological changes observed in nerve regeneration (94). Natural and synthetic enhancer-promoter (EP) systems can induce gene transcription through time, space, or environmental signals, providing a means for finely regulating expression. Constructing SCs with artificial EP promoter carrier systems to further enhance the expression intensity of pathways like Rac1/MKK7/JNK might better promote ECM regulation and peripheral nervous system regeneration. Eva Sonnenberg-Riethmacher’s team found that the neural regulatory protein ligand and its ErbB3 receptor are crucial for SC development. Defective ErbB3 expression leads to complete loss of SCs in peripheral nerve axons, muscle bundle tremors, and neuronal cell death. ECM gene periostin was significantly downregulated in ErbB3-deficient pseudo-unipolar neurons (DRG), suggesting a pathway where ECM interacts with neurons to intervene in SC gene expression (ErbB3 receptor activation-periostin gene-periostin secretion-SC migration). Feng-Chun Yang’s team, studying neurofibromas, discovered that mast cells in the ECM can infiltrate neurofibromas and secrete proteins that reshape the ECM and initiate vascularization, establishing a new interaction between Nf1−/− (homozygous mutation of the Nf1 tumor suppressor gene) SCs and Nf1+/− (heterozygous mutation of the Nf1 tumor suppressor gene) mast cells. This interaction is characterized by increased migration of Nf1+/− mast cells, closely related to the overactivation of the Ras class IA-PI3K-Rac2 pathway and significant for the formation of neurofibromas during regeneration (95).

In summary, after peripheral nerve injury conduit transplantation, the newly formed ECM will activate a series of signaling pathways through a range of growth factors, leading to complex interactions with Schwann cells. Adjusting materials and manufacturing methods, altering the physicochemical properties of ECM, and properly regulating these pathways will be crucial in promoting better recovery of structure and function after peripheral nerve injury, representing a key focus in future research in this field (Table 1).

**Table 1:** Overview of Schwann cell and extracellular matrix (ECM) interactions.

Acting factors	Routes of action/effects	References
Laminin Fibrin Type IV collagen	Promote early adhesion of SCs in two-dimensional culture, with obvious difference in adhesion ratio.	(89, 91)
Laminin	Activating NF-kappaB in Schwann cells to enhance synaptic growth.	(92)
MK/PTN gene family	Promote Schwann cell proliferation and metastasis through the SEMA3C signaling pathway, thereby promoting earlobe keloid formation.	(93)
Increase in ECM stiffness and SC diffusion	Downregulation of SC regeneration-related proteins through activation of RhoGTPase and YAP/TAZ	(94)
Cell elongation	Promotes unique SC regeneration capacity through upregulation of Rac1/MKK7/JNK, contributing to changes in cell morphology and ECM.	(94)
Neuregulin ligands and their ErbB receptors	Promote the expression of ECM gene periostin, induce the production of periostin, and promote the formation, proliferation and migration of Schwann cells.	(96)
Nf1+/− Mast cell migration	Overactivation of the Ras-like IA-PI3K-Rac2 pathway triggers the interaction between Nf1−/− Schwann cells and Nf1+/− mast cells, ultimately promoting the secretion of a type of protein that can remodel the ECM and promote angiogenesis, causing neurofibromas.	(95)

## 6 Summary and future outlook

This review has outlined one of the key focuses in the field of peripheral nerve regeneration: Schwann cells, ECM, and their intricate interactions. It is evident that the quality of the ECM environment directly determines the proliferation and developmental processes of Schwann cells, thereby influencing the prognosis of peripheral nerve regeneration after injury. By improving the materials and manufacturing processes of nerve conduits to alter the physicochemical properties of the ECM, and by supplementing with exogenous growth factors or modulating the expression of specific genes, we can enhance Schwann cell growth, proliferation, and directionality. This approach aims to achieve nerve structures that closely resemble normal states, maximizing functional recovery and overcoming the inconveniences associated with autologous nerve transplantation.

However, the effectiveness of tissue-engineered nerve conduits relying on the interplay between the ECM and Schwann cells for repairing peripheral nerve injuries is not consistently uniform. From the literature covered in this review, future prospects involve the use of collagen-SiO2-chitosan composite materials, employing electrospinning and 3D printing to mimic the intricate internal structures of natural peripheral nerves. Additionally, the injection of exogenous growth factors such as bFGF, along with the use of gene editing techniques to introduce enhancers, miRNA, and other methods to activate/silence specific genes necessary for maintaining the complex interaction between Schwann cells and the ECM, holds promise. This approach is expected to establish a comprehensive and precise medical industry chain for peripheral nerve injuries – one of the most common and challenging surgical injuries. This development could bring significant economic benefits and health improvements, profoundly impacting the fundamental research in neuroscience.

## Author contributions

MJ: Conceptualization, Funding acquisition, Project administration, Supervision, Validation, Writing – original draft, Writing – review & editing. MC: Writing – original draft, Writing – review & editing. NL: Writing – original draft, Writing – review & editing.
